# Fostering Collective Approaches in Supporting Perinatal Mental Healthcare Access for Migrant Women: A Participatory Health Research Study

**DOI:** 10.3390/ijerph19031124

**Published:** 2022-01-20

**Authors:** Kathleen Markey, Maria Noonan, Owen Doody, Teresa Tuohy, Tony Daly, Ciara Regan, Claire O’Donnell

**Affiliations:** 1 Department of Nursing and Midwifery, Faculty of Education and Health Sciences, Health Research Institute, University of Limerick, V94 T9PX Limerick, Ireland; maria.noonan@ul.ie (M.N.); owen.doody@ul.ie (O.D.); Teresa.G.Tuohy@ul.ie (T.T.); claire.odonnell@ul.ie (C.O.); 2 80:20 Educating and Acting for a Better World, St. Cronan’s National School, A98 NW42 Wicklow, Ireland; tony@8020.ie (T.D.); ciara@8020.ie (C.R.)

**Keywords:** migrant women, perinatal mental healthcare, healthcare access, solution-focused approaches, participatory health research, world café

## Abstract

Perinatal mental health is a growing public health concern. The mounting evidence examining the prevalence of perinatal mental illness identifies specific vulnerabilities and risk factors among migrant women. We know that migrant women experience persistent and systematic barriers in accessing healthcare and that healthcare services do not always respond appropriately to migrant women’s needs, highlighting the need for targeted interventions in supporting positive perinatal mental health among migrant women. The purpose of this participatory health research study was to explore perinatal mental healthcare for migrant women in Ireland, from the perspectives of a diverse range of stakeholders (healthcare service providers, community organisations/networks/associations and migrant women). A key focus of this study was to collaboratively explore solution-focused approaches to improving access to supports and healthcare services for migrant women experiencing perinatal mental illness. Following ethical approval, data were collected during three key convenings, utilising the design principles of world café philosophies. Thematic analysis led to the generation of the following two themes: *Building Capability and Capacity* and *Empowering Migrant Women*. The main conclusions lie in the provision of whole-system approaches in collectively, collaboratively and proactively planning strategies that address the many factors that affect access to healthcare services for migrant women experiencing perinatal mental illness. Drawing on the collective perspectives of a wide range of stakeholders, our innovative solution focused on providing recommendations aimed at strengthening supports and healthcare services for migrant women.

## 1. Introduction

Perinatal mental health encompasses the mental and emotional health of women throughout the perinatal period (during pregnancy and in the year after birth). During the perinatal period, women are at risk of developing an acute onset, relapse or reoccurrence of a wide range of mental illnesses [1]. One in five women in the general population will develop a mental illness during the perinatal period, and perinatal mental health is now recognised as a public health concern [2]. Untreated perinatal mental illness can have devastating effects on the health and well-being of the woman, the child and family unit [3]. Consequently, there is a renewed emphasis on the importance of early identification of perinatal mental illness through screening, diagnosing and woman/family-centred care and treatment that is timely, appropriate and responsive to the individuals’ needs.

Migrant women are particularly susceptible to experiencing a new or reoccurring mental illness during or after pregnancy [4,5,6,7]. For the purposes of this paper, the International Organisation for Migration (IOM) definition of a migrant is adopted. A migrant is defined as ‘any person who is moving or has moved across an international border or within a State away from his/her habitual place of residence, regardless of the person’s legal status, whether the movement is voluntary or involuntary, what the causes for the movement are and what the length of the stay is’ [8]. Systematic reviews examining the prevalence and risk factors of perinatal mental illness identify specific vulnerabilities among migrant women, which require consideration [7,9]. Uncertain migration status, lack of social support and low socioeconomic status are all commonly reported aspects of migration that negatively affect perinatal mental health [10]. As many as one in three migrant women experience a perinatal mental illness, illuminating the importance of ensuring supports and services are tailored to meet the needs of vulnerable women from diverse cultural, ethnic and linguistic backgrounds [9]. However, this requires developing a deeper understanding into the type of supports acceptable to migrant women experiencing perinatal mental illness and taking a proactive solution-focused approach to address service access and utilisation issues.

Ireland has experienced a rapid growth in inward migration trends over the last 20 years. In particular, the rise in new asylum-seeking applications for international protection owing to human rights violations as a result of the recent refugee crisis due to conflicts, for example in the Middle East, Asia and Africa, requires consideration. Consequently, ‘non-Irish nationals’ (a term used by the state) now represent over 13% of the population in Ireland [11], highlighting the need for healthcare services to be responsive to the needs of its patient population from diverse cultural, ethnic and linguistic backgrounds. The importance of adopting a human rights approach to healthcare delivery is a necessity [12]. Concerns exist regarding general mental health supports for migrant women living in Ireland [13]. However, there is a dearth of evidence examining the perinatal mental health supports and experiences of migrant women living in Ireland [14]. Like many other countries, there are growing reports highlighting the challenges in providing effective perinatal mental healthcare in Ireland [14,15,16,17,18]. However, these difficulties intensify for women from diverse cultural, ethnic and linguistic backgrounds. The World Health Organisation launched a Strategy and Action Plan for Refugee and Migrant Health in 2016 [19], emphasising the importance of ensuring that services and service provision are designed to meet the needs of migrant communities. However, Sweilish et al. [20], in their bibliometric analysis of global migration health research (2000–2016), highlighted the need to generate further evidence in maternal health to inform targeted interventions in practice and policy development. Therefore, there is a need to investigate and advance our understanding of pathways for improving access to services and ensuring that a proactive approach is adopted in supporting migrant women experiencing perinatal mental illness.

The international evidence acknowledges that migrant women have poorer maternal health and are generally slower at engaging with maternal healthcare services, thus highlighting the importance of focusing attention on encouraging engagement with services and supports [21,22]. A clear focus needs to address the persistent and systematic barriers to accessing perinatal mental healthcare for migrant women [23,24,25,26]. Barriers such as language proficiency [27] and cultural influences on the perceptions of perinatal mental illness and health-seeking behaviour [5,9,24] are frequently reported. However, there is limited guidance on how collective, proactive and solution-focused approaches can be developed to improve access to culturally responsive perinatal mental healthcare services and supports. This paper reports on a participatory health research approach exploring perinatal mental healthcare for migrant women in Ireland. The aim of this study was to explore the perspectives of a diverse range of stakeholders (healthcare service providers, community organisations/networks/associations and migrant women), with regard to experiences, generating solution-focused recommendations regarding supports and healthcare services for migrant women experiencing perinatal mental illness.

## 2. Research Methods

### 2.1. Research Design and Methodology

This study used a participatory health research (PHR) design in exploring perinatal mental healthcare for migrant women in Ireland, from the perspective of a diverse range of stakeholders. The benefits of incorporating PHR approaches when exploring migrant health through the lens of ‘expert by experience’ is widely acknowledged [28,29]. Incorporating PHR improves the robustness and appropriateness of research, whilst also ensuring that recommendations meet the needs of service users [30,31]. In keeping with a PHR approach, this study incorporated a soft systems analysis methodology [32], which provided a framework for three key convenings. The aim of the key convenings was to co-create a participatory forum and an interactive space for key stakeholders from different backgrounds, experiences and organisations to explore their collective roles in supporting migrant women who are at risk of or are experiencing perinatal mental illness in a structured interactive process.

The key convenings also aimed to provide opportunities to bring together stakeholders who do not usually meet each other in a group context, to connect, network and facilitate deeper forms of understanding, knowledge and information exchange, idea sharing, the consideration of diverse roles and relationship building. Adopting a ‘bottom up’ participatory approach, encouraging conversations and partnership working, support for diversity, equality, intercultural understanding and the integration of migrant women’s experiences and perceptions into the planning and development of appropriate services was fostered and promoted. In addition, empowering organisations that support migrant women experiencing perinatal mental health conditions, to define and prioritise problems systematically, develop appropriate solutions, share, and apply the knowledge generated, was critical within the approach adopted.

### 2.2. Sampling and Participants

In keeping with recommendations for planning stakeholder engagement [33], providing inclusive opportunities for a diverse range of stakeholders to meaningfully network and share experiences and perspectives was critical in the snowballing sampling approach adopted. Following ethical approval (2021_05_06_EHS), a targeted multi-stakeholder approach for the recruitment of participants was adopted in recognition of the existence of multiple areas of expertise, and of various skills and experiences that need to be brought together when exploring perinatal mental health for migrant women. A diverse range of stakeholders from a variety of settings and backgrounds were invited to participate and share the invite to others in their network, including healthcare providers, community groups, networks and associations who provide support to women and migrant communities. Participants from a diverse range of backgrounds and experiences participated in the study (n − 52), which helped in gaining a meaningful and collaborative understanding of the issues around perinatal mental health support for migrant women (see Table 1). Of the 52 participants, 26.9% (n − 14) identified themselves as migrant women and men, 19.2% (n − 10) identified themselves as NGO/community organisations/networks/associations that support migrant communities, 21.2% (n − 11) identified themselves as healthcare professionals based in hospital settings, 19.2% (n − 10) identified themselves as healthcare professionals based in community settings, and 13.5% (n − 7) identified themselves as other.

### 2.3. Data Collection

Data collection was informed by the seven integrated design principles of World Café philosophies [34]: (1) set the context; (2) create a hospitable space; (3) explore questions that matter; (4) encourage everyone’s contribution; (5) cross-pollinate and connect diverse perspectives; (6) listen together for patterns, insights, and deeper questions; and (7) harvest and share collective discoveries. The World Café method is particularly useful when exploring an area from multiple perspectives including differing perspectives with diverse community and health care stakeholders [35]. World Café discussions are informal and participatory conversations around questions that have meaning and utility for all participating and are useful for hearing and sharing differing perspectives and experiences [36].

Although data collection was originally planned face-to-face, due to COVID-19 public health restrictions, they were conducted remotely using an online platform, guided by five online hosting key decision points (KDPs) [37], to foster a feeling of safety in the virtual room and participant engagement. There were many advantages to hosting these events online, such as providing flexibility for participants and the cross-fertilisation of experiences and expertise across geographical locations in Ireland that otherwise may have been difficult to achieve. Informed written consent was obtained. Three online key convenings informed by World Café philosophies took place between August and September 2021, and each online event lasted 2.5 h. Discussions were conversational, inclusive and inviting, but were semi-structured to ensure meaning, shared understanding and collaborative dialogue around questions that matter in the real world [34]. The focus of the conversations was to collaboratively explore diverse perspectives and experiences, in identifying successes, prioritising challenges, and generating quality solutions to existing or potential problems associated with perinatal mental health for migrant women, their families and communities in Ireland.

Invited participants were randomly allocated to groups, ensuring that diverse ranges of backgrounds were represented within each group. Participants were invited to engage in three consecutive randomly selected rounds of conversations (online breakout rooms), each lasting approximately 20 min. Each breakout room discussed the same question and after each round, the participants moved to a new breakout room, until all three consecutive rounds were completed (Table 2). Throughout each discussion round, participants recorded summaries of discussions or noted key ideas in a virtual tablecloth. A graphic recorder visually documented the discussions using graphics, which were presented back to participants at the end for verification. After three consecutive rounds of conversations, a period of sharing discoveries and insights in a whole group conversation took place within a World Café harvest round. Individuals were invited to share insights from their small group conversations with the rest of the larger group as a means of sharing commonalities between conversations, developing collaborative insights and consideration of possible actions required.

### 2.4. Data Analysis

All data were pseudonymised and any names recorded were removed before data analysis commenced. Data obtained from consecutive rounds of conversations, summaries during each round of conversations and notes of key ideas recorded on virtual tablecloths and World Café harvest round were analysed. Data analysis was guided by a thematic analysis framework [38]. Thematic analysis was chosen as the best framework for data analysis, as it is systematic in its approach in identifying codes and themes created directly from the collected data. The importance of researchers immersing themselves in the data and demonstrating a methodical approach to the analysis was critical. This involved reading and re-reading the notes recorded on the virtual tablecloth, coding the data and finally developing themes. Codes were generated and grouped into clusters that formed sub-themes and themes. Through discussions within the team, the themes were reviewed, critically discussed and consensus was reached as to the final themes. Relevant extracts from notes and discussions are used to illustrate the meaning of the themes. The computer software package NVivo 12 was used to assist with data organisation, management and analysis.

## 3. Results

Data analysis led to the generation of the following two themes: building capability and capacity and empowering migrant women. Each theme is supported with sub themes explaining each theme. Building capability and capacity consists of the following sub-themes: enhancing education and training, cultivating cross agency networking and collaborative working and fostering authentic engagement. The theme empowering migrant women consists of the following sub-themes: raising awareness among migrant women, guidance on navigating services and nurturing networking opportunities and peer community supports.

### 3.1. Building Capability and Capacity

Participants consistently discussed the importance of building capabilities and capacity of services and supports in encouraging access and engagement with services. Enhancing education and training, cultivating cross agency networking and collaborative working and fostering authentic engagement are key areas identified for increasing access to services that require consideration.

#### 3.1.1. Enhancing Education and Training

It was widely acknowledged that health care professionals and community support groups overall are highly committed to meeting the needs of migrant women experiencing perinatal mental health conditions. However, the importance of providing personal and professional development opportunities for individuals working with migrant women at risk of or who are experiencing perinatal mental health needs was a key recommendation for increasing healthcare access.

*Professional development for people working in the area and utilising key stakeholders*

(Online World Café 2).

In particular, the importance of developing cultural awareness and sensitivity in understanding perinatal mental health vulnerabilities and needs for migrant women, whilst acknowledging and respecting cultural difference requires focused attention. Migrant women need to feel that they are understood and adequately supported when accessing services.

*Professionals need to be understanding of cultural difference as without understanding they can’t support*

(Online World Café 1).

Developing greater awareness of diverse cultural influences that inform perceptions of perinatal mental health and health-seeking behaviour is a necessity. Lack of awareness of cultural difference within the context of perinatal mental healthcare can present as a barrier for migrant women when accessing services and supports.

*There is a need to be aware of differing cultural practices around perinatal and postnatal care and respect for cultural norms in a scientific way for better outcomes*

(Online World Café 1).

The poignant effect that culturally informed stigmas associated with mental illness can have on perinatal mental health-seeking behaviour requires consideration when examining ways of increasing access and engagement with services. The following World Café discussion extract highlights the need to understand the individualised perceptions of mental health and mothering that impact on perceptions of perinatal mental healthcare among women from different migrant sub-groups that may have specific needs, such as refugees or asylum seekers.

*Not having the information for nurses about us, about our concerns and what we might need as refugees. It was surprising for me that they didn’t know about me and made assumptions. In my home country there is a mental health stigma; whereas here it isn’t…you mention Mental Health and we don’t speak anymore*

(Online World Café 3).

The value of experiencing proactive approaches in exploring perinatal mental health during all healthcare consultations was acknowledged as a means of providing advice and support and encouraging access to services when needed.

*I was asked about my mental health at every check-up and every call*

(Online World Café 3).

However, the assessment technique adopted needs to be meaningful, compassionate, and culturally responsive, and should encourage women to share their stories, feelings and experiences. This requires developing the courage, compassion, confidence and commitment to ask sensitive questions incorporating trauma informed screening and assessment approaches. The importance of trauma-informed care as a means of situating and understanding traumas that have contributed to women’s unique experiences and vulnerabilities is essential. Such approaches increase access to services but require specialised education and training to equip healthcare professionals with the knowledge, skills and attitudes to engage in sensitive conversations in effective ways.

*Trauma informed approach to care- women who have been through migration and associated trauma. Utilising a trauma informed approach to perinatal mental healthcare care has helped women to situate their experience within the context of that trauma and knowledge of this can provide relief*

(Online World Café 3).

#### 3.1.2. Cultivating Cross Agency Networking and Collaborative Working

Many migrant women who need perinatal mental healthcare support do not receive it, highlighting the urgent need to review existing strategies and approaches that encourage access to healthcare services and supports.

*A lot of people are falling through the gaps or {have the} potential to fall through the gaps*

(Online World Café 1).

The diverse range of supports and services available for migrant women experiencing perinatal mental illness across organisations and agencies was commended. Although it was felt that organisations and groups individually provide excellent supports and services, there was a consensus within the discussions that they often work in silos. As a result, there are limited opportunities for the cross-fertilisation of expertise and collaborative approaches in holistically supporting migrant women, which ultimately affects access to services. The need for a greater shift to working collectively both within specific organisations and across agencies as opposed to working in silos is needed as a means of improving access to healthcare services.

*The importance of sharing and defining what our roles are and informing people what each service provides and we can all be very protective of our own areas. We need to share more—there is a need for better interprofessional communication and sharing of expertise. We must not work in our silos anymore*

(Online World Café 3).

This requires developing a commitment among all healthcare and community organisations to engage in inter-agency networking opportunities as a means of creating a greater awareness of differing roles that collectively support migrant women experiencing perinatal mental illness. This can help with developing a greater understanding of the structures and services that can work together in ensuring that appropriate support is provided.

*Collective approach to perinatal mental health is needed and being more aware of the services that are available so I can refer women to these services*

(Online World Café 1).

This partnership working is critical in ensuring everybody involved in supporting migrant women in different contexts have a shared understanding of perinatal mental health and can offer guidance on appropriate supports and services available. However, this requires a collective commitment in understanding the unique vulnerabilities of migrant women and the different supports and services available.

*Create greater awareness among organisations of the need to support migrant women around the types of supports available and how to access them*

(Online World Café 2).

#### 3.1.3. Fostering Authentic Engagement

Participants consistently acknowledged the importance of developing trusting relationships and authentic engagement opportunities when communicating with migrant women experiencing perinatal mental illness. Attentively listening to the woman, understanding their unique vulnerabilities and needs, and building trust within the relationship are paramount. In particular, the importance of spending time with the women and being present is an important consideration when developing trust and authentic engagement, which encourages continued access and engagement with services.

*People owning the challenges and difficulties rebuilding the relationship and being present for the individual—people have lost trust from their origin countries so building that authenticity is essential (there are always system problems and things don’t always go to plan) but you are there to support them is important and that you can listen. Many healthcare professionals are trained ‘to do’ and not just ‘to listen’ or to confront delays and inaction in the system*

(Online World Café 2).

Creating an environment where migrant women feel a connection and are comfortable to share their experiences, feel listened to and understood is vital. This requires taking time to form connections and understand women’s experiences and concerns as a means of nurturing authentic engagement.

*Human connection—not just information and skills—attachment dynamic between someone in distress and someone responding to the woman’s distress works*

(Online World Café 3).

However, the formal healthcare setting can be daunting, and therefore informal settings and spaces where women feel comfortable in expressing their feelings in a safe environment is essential.

*Relationships with medical authorities can be daunting*

(Online World Café 3).

There were numerous examples shared where migrant women reported feeling listened to and truly supported. However, due to a lack of resources and the ‘busyness’ of healthcare settings, it can be challenging for healthcare professionals to secure the time needed to authentically engage with migrant women. Authentic engagement requires commitment but also the time and space to nurture therapeutic and trusting relationships that are necessary for encouraging access with healthcare services.

*Health care professionals only meet women for a quick snapshot—little time to spend time dialoguing with women—we need more time for health care professionals to spend time with women. Health care professionals don’t have a relationship with migrant women—so they need to be aware of the need to assess migrant women and give time and give support*

(Online World Café 1).

However, the difficulties in developing authentic engagement when there are language barriers is an important area that also requires consideration. The difficulties for women from diverse linguistic backgrounds in communicating vulnerabilities and support needs is an area of concern. The importance of ensuring access to professionally trained interpreters when required was acknowledged.

*People can slip through the cracks when an interpreter should have been used but was not. The person ends up being much sicker*

(Online World Café 3).

The importance of having interpreting services that can act as cultural mediators, translate conversations and have insights into perinatal mental health and the unique vulnerabilities of migrant women experiencing perinatal mental illness was frequently discussed across all World Café events. The education, training and follow up support for professionally trained interpreters interpreting for migrant women experiencing perinatal mental illness requires further consideration.

*The interpreter needs to have a background in the woman’s culture. Emotional distress can get lost in translation. Support for the interpreter*

(Online World Café 3).

### 3.2. Empowering Migrant Women

Despite acknowledgements of the value of the range of perinatal mental health supports for migrant women, the complexities associated with accessing and engaging with supports and services is a continued concern. Targeted interventions aimed at empowering migrant women in understanding their vulnerabilities, feelings, and support needs is one means of nurturing access and engagement with appropriate healthcare services. Raising awareness of perinatal mental health among migrant women, providing guidance on navigating perinatal mental healthcare services and supports and providing networking opportunities and peer community supports for migrant women are paramount.

#### 3.2.1. Raising Awareness among Migrant Women

Participants consistently described the complexities experienced by migrant women in accessing and engaging with healthcare services. There was general agreement across the World Café events that migrant women are often unaware of perinatal mental healthcare supports and services available and feel unsure of who to ask for guidance.

*The challenge for women to present themselves and the need for support and education and awareness of supports and services. The need for healthcare professionals to educate and inform women about services and supports*

(Online World Café 3).

Recommendations from World Café discussions highlight the importance of creating a greater awareness of perinatal mental health among migrant women, including signs and symptoms of perinatal mental illness that require interventions and of supports and services available.

*Helping women to support themselves, their wellbeing and mental health and {encouraging engagement with} wellbeing support groups is essential. Building capacity with women to help access support, reaching out to the communities and social prescribing*

(Online World Café 3).

Strategies used to raise awareness of perinatal mental health among migrant women need to be culturally responsive and made available in different languages, through different modes and platforms.

*Translated information on perinatal mental health conditions, symptoms, accessing services specific for different cultural needs and the information {should be} developed with migrant women for migrant women*

(Online World Café 1).

The importance of developing a wide range of awareness raising resources, in a variety of forms using different platforms was a re-occurring recommendation as a means of meeting the diverse needs of migrant women from different cultural, ethnic and linguistic backgrounds. Leaflets, videos and social media were some suggestions discussed.

*The need for information leaflets but these need to be in different languages to avoid language barriers*

(Online World Café 3).

*Use of videos and social media*

(Online World Café 1).

#### 3.2.2. Guidance on Navigating Services

The complexities with navigating different health care systems were identified as a particular challenge for migrant women from diverse cultural, ethnic and linguistic backgrounds, who are unfamiliar with different healthcare systems and structures. Although the range of perinatal mental healthcare supports and services were praised, the intricacies with understanding differing roles, responsibilities and systems was consistently discussed. The following World Café discussion extracts summarise the experiences and feelings of migrant women who may require perinatal mental healthcare supports.

*We are overwhelmed*

(Online World Café 3)

*A lot of people support different things… it is complicated! A directory to navigate would be helpful*

(Online World Café 2).

The difficulties in accessing healthcare services when experiencing perinatal mental health difficulties was a concern repeatedly discussed. The value of outreach services where healthcare professionals with specific knowledge and skills in supporting perinatal mental healthcare among migrant women are more visible and available in community settings was valued.

*The need for awareness and the need for somebody to talk to—the need for healthcare professionals to work more in community—have workshops and information to educate women on services and supports so they are more aware—inter-relations with people living in direct provision the need for outreach*

(Online World Café 3).

There was also a sense that the supports provided need to be geared specifically towards migrant women’s unique needs, and particular consideration needs to be given to identifying risks and vulnerabilities, whilst acknowledging the challenges for migrant women in seeking help and accessing services.

*The need for awareness of supports but also how can we encourage access and engagement and how can we ensure appropriate care. How are services being communicated and having processes in place to support. It is not enough to just have the services, we need more touch points and services needs to be so supportive—what is said needs to be communicated effectively*

(Online World Café 2).

The difficulties migrant women experience when trying to navigate perinatal mental healthcare services was widely acknowledged. ‘Knowing who to contact and how’ was a particular concern consistently discussed.

*No ideas how to access healthcare services*

(Online World Café 1).

The importance of having targeted interventions that can help migrant women to understand and navigate healthcare service structures and systems is a necessity. Having access to a directory of available services offering guidance on when to access services was one recommendation suggested.

*When we need help you often don’t know who to contact or what help you need. We need to share a database of supports available. The majority of women don’t know of the services that are available. Women are not aware of the services or who to ask for help and how to access the help. There is a need for a directory of what services are available*

(Online World Café 3).

The development of user-friendly leaflets and posters that offer guidance on when and how to access perinatal mental healthcare services was also a recommendation consistently discussed.

*We need to create flyers/posters and information so migrant women know where to access and understand how to access support and services for perinatal mental illness*

(Online World Café 2).

#### 3.2.3. Nurturing Networking Opportunities and Peer Community Supports

Although the unique identities and experiences of migrant women from diverse cultural, ethnic and linguistic backgrounds must be acknowledged, there was a consensus that migrant women experiencing perinatal mental illness do experience similar challenges when accessing healthcare services. Having opportunities for social networking and peer support when connecting with other migrant women in similar situations who are experiencing vulnerabilities and difficulties can be an excellent supportive mechanism.

*Peer support is important where women have opportunities to share perspectives and experiences with women from different cultures—this social support is important and provides social networks outside of healthcare professionals*

(Online World Café 3).

Migrant women experiencing perinatal mental illness are often hesitant to engage with healthcare professionals as a first point of contact. However, they often turn to other supports within the community, such as friends, religious leaders and community networks and associations.

*Because of language barrier 4 weeks after delivery and started to experience psychosis and reached out to a preacher*

(Online World Café 1).

The power of developing community connections where opportunities for migrant women can come together and participate in supportive and meaningful conversations was also widely acknowledged. These informal opportunities to meet other women helped to form connections and provided a ‘comfort zone’ where women could share experiences and perspectives in a safe environment.

*Informal care can’t be overemphasized…when people feel comfortable to talk at that point*

(World Café 3).

## 4. Discussion

This participatory health research highlights the complexities involved in supporting the mental and social well-being of migrant women during the perinatal period and illuminate the healthcare access difficulties experienced by migrant women. The findings reiterate results from other studies [23,25,39] in highlighting the complexities that can act as barriers to accessing healthcare at different points of the care pathway for migrant women experiencing perinatal mental illness. Although there is an urgent need to address the wider issues informing the conditions migrant women experience before, during and after migration, there is also a need to recognise the importance of achieving universal health coverage and access to quality healthcare that meet the needs of marginalised women form diverse cultural, ethnic, and linguistic backgrounds.

The findings of this study mirror those of others that report on migrant women’s hesitancy in seeking help [5,22], their unfamiliarity with healthcare systems and services [27] and the difficulties women from diverse cultural and linguistic backgrounds experience when accessing healthcare services [25,39]. This study adds to this body of knowledge as it explores solution-focused strategies from the collective perspectives of a range of stakeholders that encourage a whole-system approach to supporting migrant women experiencing perinatal mental illness. In particular, the importance of developing easily accessible supports and services that meet the needs of culturally and linguistically diverse women from sometimes complex migration conditions, is paramount. This is in keeping with Roberts et al. [27] and Heslehurst et al. [23], who highlighted the need to re-focus energies into examining ways of addressing the persistent and systematic barriers to accessing healthcare and examining why recommendations that are known to work are not adopted in practice. However, the findings of this study highlight the intricacies associated with planning such approaches, which require careful, sensitive, collective, collaborative and proactive planning. Despite the acknowledgement of the importance of effective perinatal mental health screening, concerns have been raised about approaches to screening and the use of screening tools in general perinatal mental healthcare in Ireland [17,40]. This study re-iterates the importance of proactively, collectively and comprehensively screening for perinatal mental illness during all engagement opportunities with migrant women, but the importance of doing so in culturally responsive ways requires urgent consideration. The selective and ad hoc screening approaches described highlight missed opportunities for meaningful dialogue about migrant women’s experiences and feelings. The hesitancy towards exploring sensitive topics and cultural influences informing perceptions of perinatal mental health is also an area requiring attention.

Healthcare professionals in all healthcare settings play a critical role in supporting the perinatal mental healthcare needs of migrant women and encouraging access and engagement with services and supports when required [41,42]. Lack of cultural awareness, sensitivity and knowledge, fragmented services and a lack of collaborative approaches in providing culturally responsive healthcare were the key factors contributing to missed opportunities in supporting migrant women. Healthcare professionals need to remain vigilant for particular perinatal mental health risk factors among refugee and migrant women and should ensure that appropriate supports and guidance on available services are provided in culturally responsive ways. There is a need for further education and training within this area, as the growing body of evidence reiterates healthcare professionals’ lack of knowledge and skills in providing perinatal mental healthcare in general [15,17,18].

An understanding of the convolutions of providing culturally responsive perinatal mental healthcare can support the planning of solution-focused approaches that nurture access and engagement with services when required. Education and training opportunities need to ensure that they adequately prepare healthcare professionals to respond to perinatal mental illness in culturally appropriate ways [24,42]. This requires developing a comprehensive understanding into the diverse range of cultural beliefs and norms that influence how a woman perceives perinatal mental health and their comfort with accessing services and support [10]. Opportunities to hear migrant women’s individual stories and connecting with different stakeholders can help in developing this required understanding. Developing an appreciation of a woman’s personal context including sources of stress and support structures can increase awareness of the profile of vulnerable women [43]. In addition, healthcare professionals may require further training in adapting current perinatal mental health assessments for migrant women. Culturally sensitive inquiry to identify a woman’s trauma history associated with migration is the first step to providing trauma-informed care [1]. However, the literature indicates that healthcare professionals require further training and education to prepare them to routinely ask women about past and current traumatic life events and to respond appropriately to disclosure [16,44]. Our findings indicate that women self-silence when asked about their perinatal mental health because of the stigma associated with mental health and the fears that their baby will be taken from them. The literature acknowledges that women are more likely to disclose psychological distress in the context of a trusting relationship and authentic engagement with a healthcare professional, which is more likely to develop in models of maternity care that provide continuity of midwifery care [45,46].

The diverse range of supports and healthcare services available to migrant women experiencing perinatal mental illness was commended. However, these services are fragmented and often work in silos, with limited opportunities for multi-agency networking and collaborative approaches in supporting migrant women, negatively impacting the uptake of supports and access to services. Others have also warned of the problems encountered by migrant women when services are fragmented and work in isolation [24]. The findings of this study add new insights into the complexities of fragmented services. Organisations and people who work in both healthcare and community organisations are unaware of the differing roles and responsibilities of the diverse range of supports and services available for migrant women experiencing perinatal mental illness. Therefore, their capacity and capabilities in advocating for migrant women and offering guidance on available supports and services were limited. The need for greater inter-agency communication and networking opportunities between healthcare, social care, the voluntary sector and communities was recognised as an important consideration requiring attention as a means of encouraging access and engagement with services when required. Villarroel et al. [47], in their scoping review exploring migrant health research in Ireland, reported a gap in collaborative action and advocacy among local and national organisations on migrant health issues in general, highlighting the need to examine ways of nurturing greater networking and collaboration. Opportunities for community organisations to engage in meaningful conversations, share understanding and experiences and learn about each other’s roles can help in developing a collective collaborative approach in supporting migrant women experiencing perinatal mental illness. However, it takes time, commitment, creativity and space to foster inter-agency relationships, partnership working and cross-pollination between different sectors.

The findings of this study concur with other studies reporting on the difficulties experienced by migrant women experiencing perinatal mental illness. In particular, the findings are consistent with others that report on the challenges with cross cultural communication and experiences of culturally insensitive care [5,39,41,48]. Although there are growing calls for addressing language barriers and improving the cultural appropriateness of services within maternity care settings [48,49], the challenges remain the same. Collectively, this evidence highlights the need for new ways of examining this area of practice as a means of encouraging access to and engagement with healthcare services among migrant women. This study highlights the need for developing strategies and particular interventions aimed at creating awareness of perinatal mental health and ways of navigating the diverse range of services and supports as a means of empowering migrant women to understand their vulnerabilities and feelings and gain an awareness of the different services and supports and how to access them. Providing opportunities for migrant women to understand the factors that may have contributed to their perinatal mental health issues and recognising their trauma, focusing on strength-building approaches to creating awareness, can also help to empower women. This requires firstly creating greater awareness around perinatal mental health and reducing some of the fears around perinatal mental illness, whilst collectively addressing stigmas associated with mental illness among migrant communities. Not knowing who to ask for help and the difficulties with navigating the diverse range of services and support was a consistent finding within this study. Similarly, other studies report on the difficulties migrant women experience in understanding different healthcare systems and accessing services and supports [24,39,50]. Community and social groups were seen as very valuable resources that provide a range of important supports for migrant women during and after pregnancy. Maximising opportunities for existing supportive community groups to continue their work in bringing women from diverse backgrounds together is important, whilst also considering ways of expanding such community peer supports. However, the investment in terms of time, commitment and resources requires further consideration. Examining ways of forming connections and seeking similarities of experiences, backgrounds and language can help to nurture supportive and trusting conversations for women, whilst providing an important layer of support in the community.

## 5. Strengths and Limitations

The qualitative approach to this study allowed the authors to explore stakeholders’ perspectives regarding perinatal mental healthcare for migrant women in Ireland. The study was inclusive of a variety of stakeholders from professional, support services and migrant groups. However, there are some limitations to this study, and it is necessary to recognise the influence researchers may have on the data collection and analysis process, and that the researchers’ experiences and knowledge may have influenced the interpretation of conclusions in this review. However, through reflexivity [51], the engagement of cafe facilitators and graphic artists, the researchers maintained a middle ground, meaning that “emic and etic” viewpoints were recognised and the researchers remained objective and reflexive in interpreting the findings [52,53].

## 6. Recommendations for Practice

This study highlights the importance of taking a whole-system approach to planning strategies that focus on the multifactorial aspects that affect healthcare access for migrant women experiencing perinatal mental illness. The findings re-iterate the importance of adopting a human rights-based approach to care delivery as recommended by HIQA [12]. Incorporating human rights-based approaches to perinatal mental healthcare requires planning inclusive supports and services that are easily and freely accessible to all women, whilst ensuring open, flexible and woman/family-centred approaches to care delivery that acknowledge, respect and respond appropriately to cultural differences. Education and training are required to prepare healthcare professionals to respond effectively to the specific needs of migrant women with perinatal mental illness. Such continuous professional development opportunities need to address inclusive and flexible assessment approaches, whilst acknowledging the importance of adopting perinatal mental healthcare screening that responds appropriately to women from diverse cultural, ethnic and linguistic backgrounds that may have experienced complex and difficult migration experiences. Understanding the unique vulnerabilities, experiences and feelings of migrant women experiencing perinatal mental illness whilst also appreciating the trauma and cultural influences that contribute to the experiences and perceptions of perinatal mental illness is critical. Migrant women’s input into training can support healthcare professionals’ understanding of the unique needs of migrant women. From a migrant’s perspective, anti-stigma strategies that inform women about perinatal mental health and educational opportunities that explore various community supports available to migrant women may increase access to supports. Furthermore, education and training for migrant support services, community groups and networks is recommended to inform providers of the signs of perinatal mental illness and referral pathways.

Perinatal mental health pathways need to be specific for migrant women and include referral to migrant supports in the community. Translated information ‘developed with migrant women for migrant women’ on perinatal mental health conditions, symptoms and guidance on accessing services and supports may offer a resource to increase awareness and reduce stigma associated with mental illness. Translations need to be available through a variety of formats (leaflets, posters, graphics) delivered through a variety of media platforms (print, video, social media) and available through a variety of services (e.g., direct provision facilities, maternity units, GP practices). A directory of health and social services to support migrant women to navigate healthcare services including specialist perinatal mental health services was identified as one strategy to increase access. The establishment of peer support opportunities for migrant women to share perspectives and experiences with women from different cultures requires consideration and resources.

## 7. Conclusions

Navigating healthcare services is strongly identified as a challenge for many migrant women experiencing perinatal mental illness. Despite the diverse range of available healthcare services and supports available, accessing these services remains problematic for migrant women. A lack of collaborative approaches, fragmented care and a lack of cultural awareness have led to missed opportunities for healthcare professionals in assisting this vulnerable group. Creating awareness of perinatal mental health and its often-associated stigma within differing cultural groups is the beginning of opening avenues into supporting women in this time of need. Only through education and training, developing an awareness and understanding of the wider conditions of the experiences of migrant women before, during and after migration can healthcare professionals provide culturally responsive care. Encouraging access and engagement with healthcare services for migrant women experiencing perinatal mental illness is required through building trusting relations which often begins outside of structured healthcare services such as within community support groups. Recognising the opportunities which exist within supportive community groups is invaluable, and this an area which requires further exploration in providing connections between migrant women and the services they seek. A multiagency approach is required to collaboratively unite in supporting women experiencing perinatal mental illness, allowing for the cross-pollination of supports and resources.

## Figures and Tables

**Table 1:** Demographic characteristics of participants.

Gender	Age Range	Country of Origin	Background of Participants	Number of Years Living in Ireland	Experiences of Perinatal Mental Illness
Female (n − 46) Male (n − 6)	22–29 (n − 4) 30–39 (n − 16) 40–49 (n − 26) 50–59 (n − 5) 60–65 (n − 1)	Nigeria (n − 4) Hungary (n − 1) Algeria (n − 1) China (n − 2) Ghana (n − 4) Thailand (n − 1) Somalia (n − 2) Poland (n − 2) Germany (n − 2) Albania (n − 1) Syria (n − 2) Zimbabwe (n − 2) Ireland (n − 28)	Migrant women living in Ireland (n − 12) Migrant men living in Ireland (n − 2) Representatives from NGO/community organisations that support migrant communities (n − 10) General nurse/midwife who work in hospital settings (n − 3) General nurse/midwife who work in community settings (n − 5) Perinatal mental health specialist midwives (n − 2) Mental health nurse who work in acute settings (n − 3) Social worker (n − 3) Healthcare professionals with a specific focus of supporting migrants in the community (n − 3) Community counsellors (n − 2) Linguistics/interpreter (n − 1) Doula (n − 1) Research, education, policy, practice interest (n − 5)	Up to 1 year (n − 1) 1–3 years (n − 8) 3–5 years (n − 9) Longer than 5 years (n − 34)	Personal experiences of perinatal mental illness (n − 11) Involved in supporting individuals experiencing perinatal mental illness (n − 16) Involved in caring for individuals experiencing perinatal mental illness (n − 22) No experience of perinatal mental illness or supporting/caring for an individual with perinatal mental illness (n − 3)

**Table 2:** Questions used to inform the three consecutive rounds.

Round 1 question	Share a story about when you have seen perinatal mental health services for migrant women work really well and truly be of support.
Round 2 question	In the stories you shared and heard in the last round, what made the perinatal mental health services/supports for migrant women so effective? How might we build on those things going forward?
Round 3 question	Building on what we have heard in the first two rounds, what is one step I/we might take to help bring these ideas into practice more often?

## Data Availability

The data presented in this study are available on request from the corresponding author. Data will not be shared publicly due to difficulties in the anonymization of qualitative data, as participants within this study shared their views and experiences with the assurance that their confidentiality and anonymity would be protected. Hence, the research data are not available publicly because this would compromise individual privacy and our ethical approval conditions.
